# Herbal Medicine Extracts Improve Motor Function by Anti-Inflammatory Activity in hSOD1^G93A^ Animal Model

**DOI:** 10.1155/mi/1999953

**Published:** 2025-02-13

**Authors:** Eun Jin Yang, Sun Hwa Lee

**Affiliations:** ^1^Department of KM Science Research, Korea Institute of Oriental Medicine, Yuseong-gu, Daejeon 34054, Republic of Korea; ^2^Department of Clinical Research, Korea Institute of Oriental Medicine, Yuseong-gu, Daejeon 34054, Republic of Korea

**Keywords:** amyotrophic lateral sclerosis, anti-inflammation, hSOD1^G93A^ transgenic mice, motor function, *Paeonia lactiflora* Pallas

## Abstract

Amyotrophic lateral sclerosis (ALS) is a multicomplex neurodegenerative disorder characterized by motor neuron death, muscle atrophy, and respiratory failure. Owing to its multicomplex mechanisms and multifactorial nature in the skeletal muscle and spinal cord (SC), no effective therapy has been developed. However, herbal medicines, known for their multitarget properties, have demonstrated promising efficacy with limited side effects in treating various diseases. Specifically, *Paeonia lactiflora* Pallas has been demonstrated to exhibit analgesic, antidepressant, anti-inflammatory, and neuroprotective effects. However, the pharmacological mechanisms underlying the beneficial effects of *P. lactiflora* in hSOD1^G93A^ animal models remain unexplored. Therefore, this study was conducted to investigate the multitarget effects of *P. lactiflora* in hSOD1^G93A^ transgenic mice, an ALS model. Footprint tests, western blot assays, and immunohistochemical analysis were used to assess the effect of *P. lactiflora* on the tibia anterior (TA), gastrocnemius (GC), and SC. The results revealed that *P. lactiflora* augmented motor function and decreased motor neuron loss in hSOD1^G93A^ mice. Furthermore, *P. lactiflora* significantly lowered the expression of proteins associated with inflammation and oxidative stress in the skeletal muscle (TA and GC) and SC. *P. lactiflora* also regulated autophagy function by reducing the levels of key markers, such as P62/sequestosome 1 (SQSTM1), microtubule-associated proteins 1A/1B light chain 3B, and SMAD family member 2, in the muscle and SC. Overall, *P. lactiflora* treatment improved motor function, prevented motor neuron death, and exhibited anti-inflammatory and antioxidative effects in the skeletal muscle and SC of ALS mouse models. These results suggest that *P. lactiflora* could serve as a promising multitarget therapeutic agent for systemic and multipathological diseases.

## 1. Introduction

Amyotrophic lateral sclerosis (ALS) is a neurodegenerative disease characterized by the progressive impairment of motor function and, ultimately, respiratory failure. It is linked to mutations in genes such as superoxide dismutase 1 (SOD1), TAR DNA binding protein, and fused in sarcoma (FUS) or a hexanucleotide repeat expansion in an intronic region of chromosome 9 open reading frame 71 [1]. ALS can occur in both familial and sporadic forms, with genetic mutations responsible for 10% of familial cases. Most ALS cases are sporadic, induced by environmental factors, such as trauma, pesticides, and smoking. Despite extensive research aimed at identifying efficient ALS therapies, only two drugs have been approved for treatment: riluzole, which decreases inordinate glutamate-induced toxicity, and edaravone, which attenuates oxidative stress. However, these drugs have demonstrated limited efficacy, only inhibiting disease progression and prolonging lifespan by a few months [2]. In animal models, the pathological mechanisms of ALS include neuroinflammation, mitochondrial impairment, autophagy dysfunction, protein misfolding, and protein aggregation in the spinal cord (SC) and skeletal muscles [3]. In addition, ALS can disrupt energy balance, leading to increased energy expenditure and hypolipidemia [1]. Given the multisystemic nature of ALS, the development of multitarget treatments is essential.

Neuroinflammation, driven by nonneuronal cells, including astrocytes and microglial cells, plays a key role in motor neuron death, leading to muscle paralysis and disease progression. Specifically, microglia activation results in the accumulation of reactive oxygen species, leading to neuron death and neurotoxin induction [4]. In ALS, this microglial activation is associated with the infiltration of peripheral immune cells as part of the innate immune response [5]. In addition, studies on SOD1 mice have revealed the activation of microglia, thus demonstrating the relationship between microglia activation-induced inflammatory response and disease progression [6]. Astrocyte activation has also been associated with disease progression, and pro-inflammatory cytokines have been demonstrated to induce glutamate excitotoxicity and neuroinflammation in ALS [7].

Herbal medicine has been employed to treat various diseases, including neurodegenerative conditions such as Alzheimer's and Parkinson's diseases. In a previous study, we demonstrated that an herbal combination attenuated motor neuron death by reducing inflammation and oxidative stress in an ALS animal model [8]. Specifically, *Paeonia lactiflora* Pallas has demonstrated potential in alleviating pain and reducing inflammation in neurodegenerative diseases, including Alzheimer's and Parkinson's diseases [9–11]. In an Alzheimer's disease model of neurotoxicity, paeoniflorin, an active compound of *P*. *lactiflora*, reduced inflammation by inhibiting nuclear factor kappa B and vascular endothelial growth factor signaling [12]. In addition, *P. lactiflora* has been reported to exhibit antidepressant, anti-inflammatory, and neuroprotective effects [13–15]. Both *P. lactiflora* and paeoniflorin have demonstrated inhibitory effects on polyglutamine-medicated aggregations, suggesting that *P. lactiflora* may be a valuable candidate for treating other polyglutamine diseases [16]. However, the pharmacological mechanisms underlying the beneficial effects of *P. lactiflora* in hSOD1^G93A^ animal models have not been evaluated. Therefore, this study was conducted to investigate the multitarget effects of *P. lactiflora* on motor function in an ALS mouse model. We found that *P. lactiflora* improved motor function and inhibited motor neuron death in the muscles and SC via anti-inflammatory, antioxidative, and autophagy regulatory activity in an ALS animal model. The results of this study may provide valuable insights into the potential of *P. lactiflora* in improving motor activity in movement disorders such as ALS.

## 2. Materials and Methods

### 2.1. Animals

hSOD1^G93A^ mice were purchased from Jackson Laboratory (Bar Harbor, ME, USA) and maintained as described previously [17]. The experimental protocol for this study was approved by the Institutional Animal Care and Use Committee of the Korea Institute of Oriental Medicine (protocol number: #17-061). All mice were housed under standard conditions, provided free access to food and water, and maintained at a constant temperature of 20–23°C, a humidity level of 50% ± 10%, and a 12/12 h light/dark cycle.

### 2.2. Preparation of *P. lactiflora* Extracts and Treatment

*P. lactiflora* was purchased from Kwangmyungdang Medicinal Herbs Co. (Ulsan, Republic of Korea). *P. lactiflora* was subjected to an extraction process using distilled water, followed by filtration and concentration of the water-based extracts under reduced pressure. Subsequently, the final products were freeze-dried to obtain powdered extracts, which were stored at −20°C until further use. Prior to administration, the pulverulent *P. lactiflora* extracts were reconstituted in distilled water.

The 8-week-old male mice were randomly divided into three groups: nontransgenic mice (nTg, *n* = 8), transgenic hSOD1^G93A^ mice (Tg, *n* = 8), and *P. lactiflora* extract-administered transgenic mice (PL + Tg, *n* = 8). *P. lactiflora* extract was administered orally at a dose of 300 mg/kg once-daily to the hSOD1^G93A^ transgenic mice for 6 weeks.

### 2.3. Footprint Test

Footprint tests were conducted the day before mice were sacrificed to assess the level of motor function [18, 19]. The hind paws of mice were coated with nontoxic ink, and the mice were guided through an alley measuring 70 cm in width, 16 cm in length, and 6 cm in height. This experiment was performed at least three attempts to obtain clear and distinguishable footprints [19]. Moreover, this experiment was conducted with a blinded group design.

### 2.4. Tissue Preparation

The tibia anterior (TA) and gastrocnemius (GC) muscles, along with the SC of 14-week-old mice, were collected after euthanasia via intraperitoneal injection of avertin (250 mg/kg). The tissues were perfused with phosphate-buffered saline (PBS) and homogenized in radioimmunoprecipitation assay buffer (50 mM Tris–Cl [pH 7.4], 1% NP-40, 0.1% sodium dodecyl sulfate [SDS], and 150 mM NaCl), a protease inhibitor, and phosphatase inhibitor cocktail (Thermo Fisher Scientific, Waltham, MA, USA). After centrifugation at 13,000 rpm for 15 min at 4°C, the protein concentrations of the homogenized tissues were quantified using a BCA assay kit (Thermo Scientific).

### 2.5. Western Blot Analysis

Tissue supernatants denatured with SDS sample buffer were separated using SDS-polyacrylamide gels and transferred to polyvinylidene fluoride (PVDF) membranes. To assess the expression of target proteins, the PVDF membranes were blocked with tris-buffered saline/5% skim milk (Sigma, St. Louis, MO, USA) and subsequently incubated overnight at 4°C with various primary antibodies: heme oxygenase 1 (HO1), ferritin, and tubulin (all at 1:1000; Abcam, Cambridge, MA, USA); transferrin and actin (all at 1:1000; Santa Cruz Biotechnology, CA, USA); p62 and microtubule-associated protein 1A/1B light chain 3B (LC3B) (all at 1:1000; Cell Signaling Technology, Danvers, MA, USA); and glial fibrillary acidic protein (GFAP) (1:5000; Agilent Technologies, Santa Clara, CA, USA). Proteins were detected using an enhanced chemiluminescent substrate (SuperSignal West Femto, Thermo Scientific), and immunoblotted bands were visualized using a ChemiDoc image analyzer (Bio-Rad, Hercules, CA, USA).

### 2.6. Immunohistochemistry

Immunohistochemistry was performed as described previously [20]. Briefly, SC tissues were extracted and fixed with 4% paraformaldehyde after mice were sacrificed. The fixed tissues were embedded in paraffin and sectioned for staining. The sectioned SC tissues were deparaffinized using serial ethanol and xylene. Next, the deparaffinized tissues were immunostained with primary antibodies, including anti-ionized calcium-binding adaptor molecule 1 (Iba1) (Fujifilm Wako Chemical, Richmond, VA, USA), anti-GFAP (Agilent Technologies, Santa Clara, California, United States), and anticholine acetyltransferase (ChAT) (Thermo Scientific, Wilmington, DE, USA), for 12 h. After washing the SC slices with PBS, the slides were incubated with secondary antibodies (antimouse IgG and antirabbit IgG). Following another round of washing, the antibody complexes were detected using the Vectastain ABC kit (Vector Laboratory Inc. Burlingame, CA, USA) and the DAB kit (Vector Laboratory Inc.). Subsequently, the stained slides were examined using an Olympus camera (DP72; Tokyo, Japan) at an original magnification of 200×. The number of positively stained cells was counted in three alternating sections from each blinded SC sample.

### 2.7. Statistical Analysis

Data were evaluated using a one-way analysis of variance and Tukey's multiple comparison test. Analyses were performed using GraphPad Prism 5.0 (GraphPad Software, San Diego, CA, USA), and data are presented as the mean ± standard error of the mean. Statistical significance was set at *p* < 0.05.

## 3. Results

### 3.1. *P. lactiflora* Improved Motor Activity in hSOD1^G93A^ Mice

**Figure 1:**
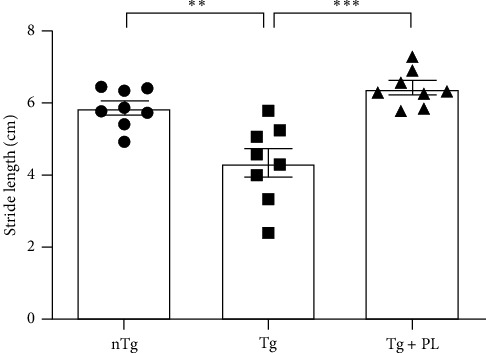
*P. lactiflora* treatment improved motor function in hSOD1^G93A^ mice. After *P. lactiflora* administration, motor function was assessed using footprint comparisons between groups. Data are expressed as the mean ± standard error of the mean (*n* = 8, *⁣*^*∗∗*^*p* < 0.01, *⁣*^*∗∗∗*^*p* < 0.001). Statistical analysis was performed as described in Section 2. nTg, nontransgenic mice; Tg, hSOD1^G93A^ mice; Tg + PL, *P. lactiflora* extract-treated hSOD1^G93A^ mice.

Given that symptomatic hSOD1^G93A^ mice exhibit decreased motor function, we investigated the effect of *P. lactiflora* on motor activity in this mouse model. To assess motor function, hSOD1^G93A^ mice were subjected to footprint tests after the administration of *P. lactiflora* (300 mg/kg) for 6 weeks. The motor function of hSOD1^G93A^ mice was reduced by 1.3-fold compared with that of age-matched nTg mice (Figure 1). However, *P. lactiflora* treatment improved motor activity by 1.4-fold, resulting in stride lengths of 6.4 ± 0.5 cm in hSOD1^G93A^ mice compared with that in Tg mice (Figure 1). These results suggest that *P. lactiflora* administration improved motor function in hSOD1^G93A^ mice.

### 3.2. *P. lactiflora* Reduced the Expression of Oxidative Stress-Related Proteins in hSOD1^G93A^ Mice

**Figure 2:**
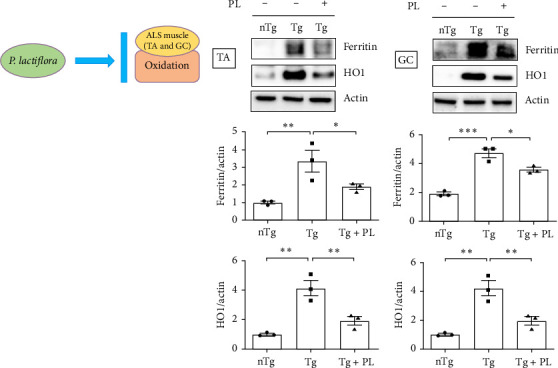
*P. lactiflora* reduced the levels of oxidative stress-related proteins in the TA and GC muscles of ALS mice. Immunoblots of each group of mice are depicted: (nTg, *n* = 3), hSOD1^G93A^ mice (Tg, *n* = 3), and *P. lactiflora* extract-treated hSOD1^G93A^ mice (Tg + PL, *n* = 3). (A) Representative data for the western blot analysis and quantification of ferritin and HO1 in the TA of each group. (B) GC tissues were immunoblotted with ferritin and HO1 in nTg, Tg, and Tg + PL mice (*n* = 3). Quantification of the immune blots was conducted using actin as a loading control. Statistical analysis was performed as described in Section 2 (*⁣*^*∗*^*p* < 0.05, *⁣*^*∗∗*^*p* < 0.01, *⁣*^*∗∗∗*^*p* < 0.001). ALS, amyotrophic lateral sclerosis; GC, gastrocnemius; HO1, heme oxygenase 1; nTg, nontransgenic mice; PL, *P. lactiflora*; TA, tibia anterior; Tg, hSOD1^G93A^ mice; Tg + PL, *P. lactiflora* extract-treated hSOD1^G93A^ mice.

To investigate the effect of oxidative stress in the muscle and SC of hSOD1^G93A^ mice after *P. lactiflora* (300 mg/kg) treatment, we examined the expression of oxidative stress-related proteins, including ferritin and HO1, in the TA, GC, and SC of hSOD1^G93A^ mice. Quantitative immunoblotting results revealed that the expression of ferritin and HO1 increased by 3.3- and 4.2-fold, respectively, in the TA of the Tg group compared with that in the nTg group (Figure 2A). However, *P. lactiflora* administration led to significant reductions in expression (1.8- and 2.1-fold) in the Tg + PL group. In addition, the levels of ferritin and HO1 proteins were increased by 2.5- and 4.2-fold, respectively, in the GC of the Tg group compared with that in the nTg group. However, after 6 weeks of *P. lactiflora* administration, significant decreases (1.3- and 2.1-fold) were observed in the Tg + PL group (Figure 2B).

**Figure 3:**
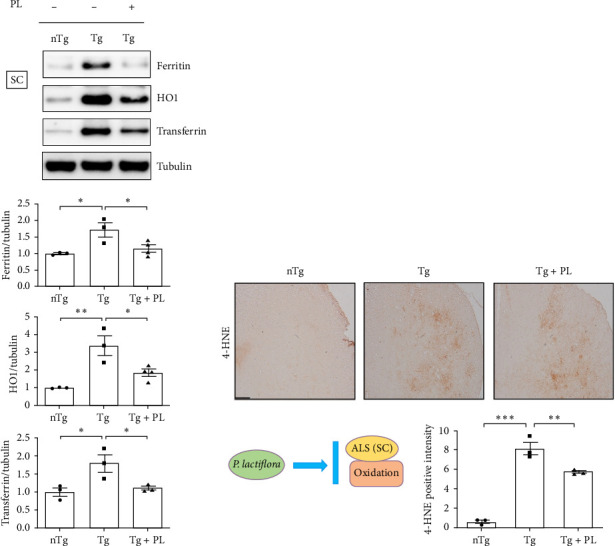
*P. lactiflora* attenuated the levels of oxidation-related proteins in the SC of hSOD1^G93A^ mice. (A) Representative data for immune blots of ferritin, HO1, and transferrin in the SC (*n* = 3). Quantification of the positive intensity of ferritin, HO1, and transferrin in each image. (B) Representative images of immunohistochemistry with anti-4-HNE in the SC in each group, scale bar = 50 μm. Quantification of the positive intensity of 4-HNE in each group. Statistical analysis was performed as described in Section 2 (*⁣*^*∗*^*p* < 0.05, *⁣*^*∗∗*^*p* < 0.01, *⁣*^*∗∗∗*^*p* < 0.001). 4-HNE, 4-hydroxynonenal; HO1, heme oxygenase 1; nTg, nontransgenic mice; PL, *P. lactiflora*; SC, spinal cord; Tg, hSOD1^G93A^ mice; Tg + PL, *P. lactiflora* extract-treated hSOD1^G93A^ mice.

Additionally, in the SC, the expression levels of oxidative stress-related proteins, including ferritin, HO1, and transferrin, were increased by 1.7–, 3.4-, and 1.8-fold, respectively, in Tg mice compared with those in nTg mice (Figure 3A). However, *P. lactiflora* treatment reduced the levels of ferritin, HO1, and transferrin by 1.5–, 1.8-, and 1.5-fold, respectively, in the SC of hSOD1^G93A^ mice compared with those in Tg mice. Similarly, the expression of 4-hydroxynonenal (4-HNE) was significantly increased by 13.6-fold in the SC of Tg mice compared with that in nTg mice; however, *P. lactiflora* treatment decreased this expression by 1.4-fold compared with that in Tg mice (Figure 3B).

### 3.3. *P. lactiflora* Attenuated Motor Neuron Death in the SC of hSOD1^G93A^ Mice

**Figure 4:**
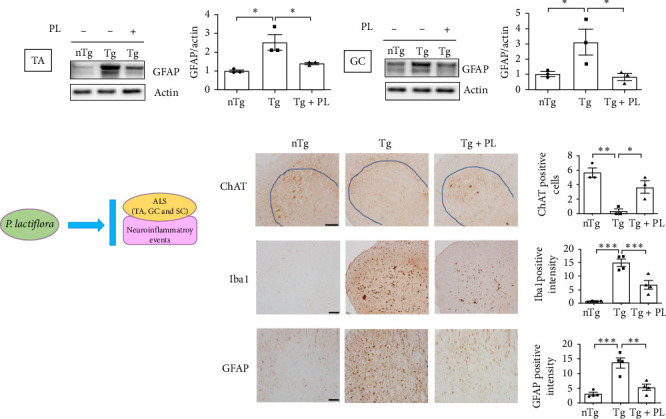
*P. lactiflora* prevented motor neuron loss and enhanced antineuroinflammatory effects in the anterior horn of the SC in hSOD1^G93A^ mice. Representative data for the western blot analysis of GFAP in the TA (A) and GC (B) of hSOD1^G93A^ mice. Quantitative analysis of the expression levels of GFAP normalized to those of actin. (C) Representative images of ChAT, Iba1, and GFAP in the anterior horn of the SC of hSOD1^G93A^ mice. Quantification of the positive intensity of ChAT, Iba1, and GFAP in each image. Statistical analysis was performed as described in Section 2 (^∗^*p* < 0.05, ^∗∗^*p* < 0.01, and *⁣*^*∗∗∗*^*p* < 0.001). ChAT, choline acetyltransferase; GC, gastrocnemius; GFAP, glial fibrillary acidic protein; Iba1, ionized calcium-binding adaptor molecule 1; nTg, nontransgenic mice; PL, *P. lactiflora*; SC, spinal cord; TA, tibia anterior; Tg, hSOD1^G93A^ mice; Tg + PL, *P. lactiflora* extract-treated hSOD1^G93A^ mice.

Given that ALS is characterized by motor neuron death, we investigated the effect of *P. lactiflora* on motor neuron survival in the SC of Tg mice. GFAP expression was increased by 2.5-fold in the GC of Tg mice compared with that in nTg mice; however, *P. lactiflora* treatment reduced this expression by 2.6-fold in Tg + PL mice compared with that in Tg mice (Figure 4A). Similarly, *P. lactiflora* treatment attenuated the levels of GFAP (by 3.8-fold) in the GC of Tg mice compared with that in Tg mice (Figure 4B). In addition, the positive intensity of Iba1 and GFAP in the SC was significantly reduced by 2.2- and 2.5-fold, respectively, in the *P. lactiflora*-treated mice compared with that in Tg mice (Figure 4C). Furthermore, *P. lactiflora* treatment dramatically protected motor neurons, as evidenced by anti-ChAT staining, resulting in a 2.2-fold increase in staining intensity in the SC of Tg mice (Figure 4C).

### 3.4. *P. lactiflora* Ameliorated Autophagy Dysfunction in hSOD1^G93A^ Mice

**Figure 5:**
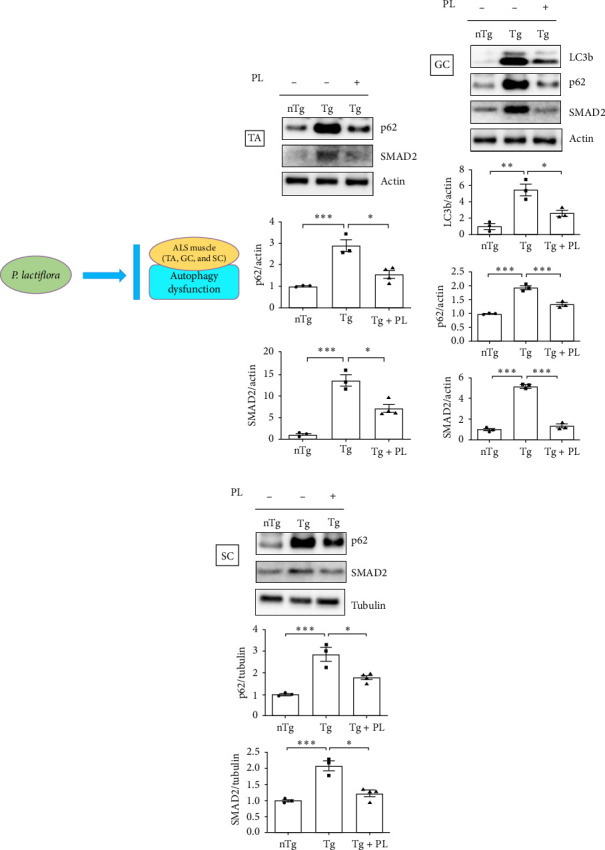
*P. lactiflora* regulated autophagy function in the muscles and SC of hSOD1^G93A^ mice. Representative western blot images displaying the expression of p62 and SMAD family member 2 (SMAD2) in the TA (A), microtubule-associated protein 1A/1B light chain 3B, p62, and SMAD2 in the GC (B), and p62 and SMAD2 in the SC (C) of each mouse group. The quantification of each blot was normalized with actin or tubulin. Statistical analysis was performed as described in Section 2 (*⁣*^*∗*^*p* < 0.05, *⁣*^*∗∗*^*p* < 0.01, and *⁣*^*∗∗∗*^*p* < 0.001). GC, gastrocnemius; nTg, nontransgenic mice; PL, *P. lactiflora*; SC, spinal cord; TA, tibia anterior; Tg, hSOD1^G93A^ mice; Tg + PL, *P. lactiflora* extract-treated hSOD1^G93A^ mice.

Crippa et al. [21] demonstrated that the autophagic markers LC3B and p62 are associated with disease progression in the skeletal muscle and SC of ALS mice. To determine the effect of *P. lactiflora* on autophagy function, we examined the expression levels of these autophagy markers in the muscle and SC of nTg, Tg, and Tg-PL mice. In the TA, the expression of P62 was decreased by 1.9-fold in the Tg + PL group compared with that in the Tg group (Figure 5A). In the GC, the increased levels of LC3B and p62 proteins in Tg mice were significantly reduced by 2.1- and 1.5-fold, respectively, in Tg + PL mice (Figure 5B). Similarly, *P. lactiflora* treatment reduced the levels of P62 in the SC of Tg mice by 1.6-fold compared with that in saline-treated Tg mice (Figure 5C). Nakamura et al. [22] demonstrated that a dysfunctional transforming growth factor–beta–Smad signal transduction pathway may play a role in the pathogenesis of ALS. In our study, the expression level of SMAD2 was increased in the TA, GC, and SC of hSOD1^G93A^ mice compared with that in nTg mice. Notably, SMAD2 expression was reduced by 1.9–, 4.0-, and 1.7-fold in the TA, GC, and SC of Tg + PL mice compared with that in Tg mice, respectively (Figure 5).

## 4. Discussion

ALS is an incurable neurodegenerative disease characterized by motor neuron death and muscle atrophy. Several studies have been conducted in an attempt to identify treatments for ALS. Although riluzole and edaravone have been used as clinical therapies, they offer limited effectiveness in extending survival. Therefore, there are currently no effective drugs available for treating patients with ALS. Given that ALS is a multifactorial disease, the development of drugs that can target multiple pathways simultaneously is essential.

The release of pro-inflammatory and anti-inflammatory factors can have both harmful and protective effects on the host. Key players in the immune system, including macrophages in muscles, microglial cells in the nervous system, and B and T cells, are essential for host protection. Notably, ALS is induced by an increase in the levels of inflammatory and oxidative factors in the SC and muscle. Abnormal immune responses and an increase in oxidative stress are also critical factors in neurodegenerative diseases, including ALS, leading to the loss of motor neurons and muscle paralysis. Within the brain, nonneuronal cells, such as microglia and astrocytes, are involved in immune responses. An abnormal increase in astrogliosis and microgliosis levels results in motor neuron degeneration and cell death in both patients with ALS and ALS animal models [23]. In addition, motor neuron degeneration leads to inflammation in the skeletal muscle, with increases in the expression of glial markers, such as GFAP, p75 neurotrophin receptor, and S100*β*, which are associated with ALS pathology [24]. hSOD1^G93A^ mice have been used as ALS animal models to investigate the etiology and pathogenesis of familial ALS caused by SOD1 mutations [25]. Moreover, most research on microgliosis and astrogliosis in ALS has primarily been conducted using hSOD1^G93A^ mice [26, 27]. Furthermore, an in vitro coculture system with mutant SOD1 astrocytes and neurons demonstrated that mutant SOD1 astrocytes release toxic substances that lead to motor neuron death [28]. This finding suggests that astrocytes play a role in motor neuron survival within a noncell autonomous environment in ALS. In addition, the levels of pro-inflammatory cytokines, such as type I interferon, were observed to be elevated in astrocytes, correlating with motor neuron death in the SC of SOD1 ALS mice [29]. In ALS, both muscle and peripheral nerve pathologies are involved in the loss of motor neuron connections at the neuromuscular junction [30]. Hegedus, Putman, and Gordon [31] reported an increase in the levels of macrophages in the nerve fibers and intramuscular axons of ALS mice, along with activated macrophages in the neuromuscular junctions of ALS animal models [32]. In patients with ALS, immune system disruptions, including an increase in CD4+ cell numbers and a decrease in T-cell levels, have been associated with clinical pathology and disease progression [33].

In both animal models of ALS and human patients with ALS, oxidative stress levels are increased in the SC, motor cortex, and cerebrospinal fluid, although it remains unclear whether this increase causes multiple pathologies, such as lipid peroxidation, or the pathological events lead to an increase in oxidative stress. Several antioxidants have been investigated as potential treatments for patients with ALS. For example, edaravone, a free radical scavenger, has been accepted by the FDA for the treatment of patients with ALS to help delay disease progression. However, its effect on extending the survival of patients with ALS is limited. In addition, although other antioxidants, such as acetylcysteine and creatine, have demonstrated efficacy in ALS animal models, they have not proven effective in improving survival or delaying disease progression in patients with ALS [34].

Oxidative stress is closely associated with mitochondrial dysfunction and occurs in the context of RNA dysmetabolism. For example, oxidative stress induced by arsenite can lead to RNA processing alterations, which results in the mislocalization of TAR DNA-binding protein 43 (TDP43) from the nucleus to the cytoplasm and the formation of aggregates [35]. In SOD1^G93A^ mice, mitochondrial dysfunction appeared to affect mitochondrial axonal transport, and DNA/RNA binding proteins, such as TDP43 and FUS, induced mitochondrial alternation and decreased the activity of the respiratory complex. Consequently, the activation of forkhead box O3a resulted in the downregulation of the expression of nuclear-encoded genes involved in mitochondrial function [36].

In this study, *P. lactiflora* treatment significantly reduced the observed increase in the levels of microglial cells and astrocytes in the SC of hSOD1^G93A^ mice. In addition, the expression of oxidative stress-induced proteins was dramatically attenuated in the GC and TA muscles and SC of *P. lactiflora*-treated hSOD1^G93A^ mice. These results suggest that *P. lactiflora* could serve as an effective anti-inflammatory and antioxidative therapeutic agent for neurodegenerative diseases.

Muscle atrophy is induced by inflammation and autophagy dysfunction [37]. ALS-related mutations in genes, such as TANK-binding kinase 1, optineurin (OPTN), sequestosome 1 (SQSTM1), and valosin-containing protein, lead to autophagy dysfunction, which in turn activates microglial function and the innate immune response [38]. The mutant G93A SOD1 accelerates the association with OPTN and is involved in OPTN-mediated mitophagosome formation and mitophagy flux [39]. Additionally, SOD1 aggregates are recognized by SQSTM1/p62, a ubiquitin-binding scaffold protein that interacts with LC3 on autophagosome membranes. Furthermore, misfolded proteins can impair nuclear protection against oxidative stress, downregulate RNA metabolism [40, 41], and lead to the development of abnormal mitochondria, such as paracrystalline inclusions and abnormal cristae, in the skeletal muscles of patients with ALS [42]. These findings suggest that metabolic dysfunction plays a role in ALS. Aggregates of mutant SOD1 inhibit the function of voltage-dependent anion channel 1, disrupting the exchange of ATP for ADP and leading to mitochondrial dysfunction [43]. TDP43 also accumulates in the mitochondria, leading to a decrease in complex I activity [44].

In a previous study, we discovered that herbal medicine improves mitochondrial function and inhibits the disruption of mitochondrial cristae structure in hSOD1^G93A^ mice [8]. Therefore, we hypothesized that the decrease in the expression of oxidative stress-related proteins after *P. lactiflora* treatment will regulate mitochondrial respiratory function in the ALS model. Although this study demonstrated that *P. lactiflora* treatment regulated autophagy function, further research is necessary to identify the specific bioactive compounds involved in regulating the autophagy signaling pathway, including the mammalian target of rapamycin inhibitors.

## 5. Conclusions

This study demonstrated that *P. lactiflora* treatment improved motor function and prevented motor neuron loss in an ALS mouse model. In addition, our findings suggested that the molecular mechanisms underlying the effects of *P. lactiflora* are attributed to its anti-inflammatory and antioxidative effects on the skeletal muscle and SC of hSOD1^G93A^ mice. Specifically, *P. lactiflora* administration reduced the levels of GFAP, HO1, 4-HNE, transferrin, and ferritin in the muscle and SC of hSOD1^G93A^ mice. Overall, *P. lactiflora* could serve as a potential treatment option for diseases caused by various pathological factors, including ALS. However, before *P. lactiflora* can be considered for the treatment of patients with ALS, further investigation into its effects on survival rates across various ALS animal models is necessary. Additionally, studies examining the mechanisms underlying the effects of *P. lactiflora* on motor function and neuroprotection should be conducted in patients with ALS. To develop a *P. lactiflora*-based drug for ALS treatment, it is essential to first identify the bioactive compounds associated with antioxidative and anti-inflammatory functions in *P. lactiflora*.

## Data Availability

The data that supports the findings of this study are available in the article.
